# “You’ve got to settle down!”: Mothers’ perceptions of physical activity in their young children

**DOI:** 10.1186/s12887-015-0466-9

**Published:** 2015-10-08

**Authors:** Megan H. Pesch, Erin E. Wentz, Katherine L. Rosenblum, Danielle P. Appugliese, Alison L. Miller, Julie C. Lumeng

**Affiliations:** Division of Developmental and Behavioral Pediatrics, Department of Pediatrics and Communicable diseases, University of Michigan, 1500 Medical Center Drive, Ann Arbor, MI 48109 USA; School of Kinesiology, Center for Physical Activity and Health in Pediatric Disabilities, University of Michigan, 1402 Washington Heights, Ann Arbor, MI 48109 USA; Center for Human Growth and Development, University of Michigan, 300 North Ingalls Street 1031 NW, Ann Arbor, MI 48109-0406 USA; Appugliese Professional Advisors, LLC, 5 Piece Way, North Easton, MA 02356 USA; School of Public Health, Department of Health Behavior and Health Education, University of Michigan, 3718 School of Public Health 1, 1415 Washington Heights, Ann Arbor, MI 48109-2029 USA; Center for Human Growth and Development, University of Michigan, 300 North Ingalls Street 1034 NW, Ann Arbor, MI 48109-0406 USA

## Abstract

**Background:**

Mothers are important mediators of children’s physical activity (PA) level and risk of obesity, however previous studies of maternal perceptions of child PA have been limited. Furthermore, it is unknown if maternal perceptions of child PA are predicted by family, mother and child characteristics. Therefore objectives of this study were to 1) evaluate maternal perceptions of PA in their children and 2) test associations of family, mother and child characteristics with these perceptions.

**Methods:**

278 low-income mothers of children (mean age 70.9 months) participated in an audio-taped semi-structured interview. Transcripts were systematically analyzed using the constant comparative method and themes were generated. A coding scheme to classify the themes appearing in each transcript was developed and reliably applied. Anthropometrics were measured. Demographics and questionnaires (the Confusion, Hubbub and Order Scale, The Parenting Scale, and the Child Behavior Questionnaire (CBQ)) were collected. Logistic regression models were used to test the associations of family, mother and child characteristics with each theme.

**Results:**

In this sample of low-income United States mothers, two themes emerged: 1) Mothers perceive their children as already very active (87.8 %, n = 244), predicted by the child being younger, the child not being overweight, and higher child CBQ Activity Level; and 2) Mothers view their children’s high activity level as problematic (27.0 %, n = 75), predicted by lower Parenting Laxness, the child being male and lower child CBQ Inhibitory Control.

**Conclusions:**

Low-income United States mothers have unique perceptions of PA in their children; these beliefs are associated with characteristics of the child and mother but not characteristics of the family. Further understanding of contributors to maternal perceptions of child PA may inform future childhood obesity interventions. The influence of these perceptions on physical activity outcomes in low-income children should be pursued in future research.

## Background

Engaging children in physical activity (PA) can lower their risk for obesity [1]. Mothers, in particular, have been cited as important mediators of childhood obesity prevention efforts, as they play a central role in shaping their children’s activity level [2]. The American Academy of Pediatrics [3, 4] recognizes the significance of parental influence on children’s activity, recommending that pediatric providers counsel parents on the importance and promotion of PA in their children at each visit. Mothers’ parenting behaviors around PA have been cited as an area of focus for child obesity prevention strategies [5], however, little is known about how pediatric providers can successfully engage and motivate mothers to support PA in their children [6]. Thus, a better understanding of maternal perceptions of child PA would inform the careful development of effective prevention and intervention strategies [7].

Parental perceptions of PA in young children have been explored primarily through qualitative analyses of focus groups with parents [2, 8–12]. This literature has generally focused on parent-identified barriers and contributors to child PA [9, 10, 13], with few studies [2, 14, 15] examining deeper perceptions, motivations and/or beliefs. Parents reported that while they believed they themselves are important contributors to shaping their young children’s PA habits [8, 10, 15, 16], they also believed that children’s PA levels are shaped by innate characteristics of the child such as sex [8], age [10], personality [8, 16] and child preferences [9, 10, 12, 13]. They noted being generally satisfied with their own child’s PA level [10, 16], but also described a number of barriers to promoting PA such as being a single parent [9], high cost [10, 11, 13, 16, 17], lack of facilities and organized activities [9, 13], safety concerns [7, 9, 10, 13], and insufficient time or busy schedules [7, 9–11, 16, 17].

The current study sought to fill several gaps in this literature. First, much prior work has been conducted using focus groups [7–12], which may lead to bias if participants agree with the group rather than offering personal opinions [18]. Second, studies that have used interviews have had relatively small [13, 16, 17, 19], non-ethnically/racially diverse [13, 17] samples or have focused only on mothers of infants and toddlers [19] or mothers of girls only [13]. Third, prior work has primarily been conducted with Australian, European and Canadian cohorts [7–11, 16, 19] and it is unclear if results are generalizable to United States (US) mothers.

The objectives of the study were two-fold. First, we sought to identify maternal beliefs, goals and perceptions of PA in their young children in an English-speaking low-income US population through a semi-structured interview. Second, we sought to determine if themes identified through maternal responses are associated with family, mother or child factors.

## Methods

### Participants

Between 2009 and 2011, female primary caregiver-child dyads were recruited into a longitudinal cohort from Head Start programs in Southeastern Michigan with some (*n* = 17) additional dyads recruited in May 2013 by flyers distributed to Head Start locations. A total of 278 female primary caregiver-child dyads participated. Of the female primary caregivers, 95 % were biological mothers, while the remaining 5 % were adoptive mothers, stepmothers and grandmothers; we refer to the entire group as “mothers.” Families participated in a study described to them as investigating associations between stress and eating in children in 2009–2011. Of the original cohort of 380 dyads, 85 % were contacted by phone in 2011–2013 and invited to participate in this follow-up study, which was described as a research study to understand the different ways mothers feed children. A total of 15 % were lost to follow up or deemed to not be eligible for this follow up study. Of those who were contacted, 92 % agreed to participate in this follow up study. The sample was further limited to those with complete data, resulting in a final sample size of 278. Eligible mothers were fluent in English and had less than a college degree. Exclusion criteria for the mothers included serious food allergies or any form of disordered eating. Written informed consent was obtained from the mothers. Mothers were compensated $100 for their participation. The study was approved by the University of Michigan Institutional Review Board.

For the children, exclusion criteria consisted of a gestational age less than 35 weeks, significant perinatal or neonatal complications, serious medical problems or food allergies, any form of disordered eating or being in foster care. Because all child participants were originally recruited from Head Start programs, they were at the time of recruitment into the original study aged three- to four-years and living in low-income families. Characteristics of the sample are shown in Table 1.

**Table 1:** Participant characteristics (*N* = 278)

Participant characteristics	
	Mean (SD, range) or n (%)
Family characteristics	
Single parent household	124 (44.6 %)
Total family CHAOS score	4.0 (3.2, 0.0–12.0)
Mother characteristics	
Maternal race/ethnicity:	
White non-Hispanic	194 (69.8 %)
Other	21 (7.4 %)
Mother is overweight (BMI ≥ 25)	214 (77.0 %)
Parenting Laxness score	2.7 (1.0, 1.0–5.9)
Employment status (employed vs. not)	166 (59.7)
Child characteristics	
Child age (months)	70.9 (8.3, 48.3–96.8)
Child sex (male)	145 (52.2 %)
Child is overweight (BMI ≥ 85^th^ %)	112 (40.3 %)
Child temperament:	
CBQ Activity Level	5.2 (1.0, 2.3–7.0)
CBQ Attentional Focusing	4.8 (1.1, 1.3–7.0)
CBQ Inhibitory Control	4.6 (1.1, 1.7–7.0)
CBQ Impulsivity	4.9 (1.0, 2.0–7.0)

### Overall study procedure

This study consisted of two study visits, which were conducted either in the participant’s home or at a local community center, depending on the mother’s preference. During the first study visit, the mothers completed questionnaires and participated in a semi-structured interview with a trained interviewer with the child not present. During the second study visit, which occurred 4.00 (+/− 5.52 SD; range 0 to 47) days later, mother and child anthropometrics were measured. All data were collected by bachelors’ level research assistants who did not participate in the subsequent qualitative analysis.

### Semi structured interview

Interviews included 45 open-ended questions that were designed to elicit maternal narratives about feeding and PA. The interview guide has been published elsewhere [20]. The methodology of development and piloting of the interview have been published elsewhere [21, 22]. Interviewers were trained to avoid giving positive, negative, or leading reactions to mother’s answers. The present report only describes the analysis of responses to four questions (Table 2) embedded within the interview that occurred after a set of questions about the family’s mealtimes, maternal feeding practices, and television-viewing habits.

**Table 2:** Questions from the semi-structured interview

How would you describe your child’s activity level?	
Do you have any concerns about your child’s activity level?	
How is it similar or different from your own?	
Do you do anything to help change it? How does that work?	

Interviews were transcribed verbatim by research assistants. Transcripts were systematically analyzed using the constant comparative method [23], an analytic approach in which the researcher reads each transcript, carefully noting possible themes, and compares the themes with those already identified in prior transcripts. As each additional transcript is read, the researcher considers whether the transcript contains previously identified themes, or if a new theme has emerged. The researcher typically begins by generating a large number of themes, but some are eventually discarded as infrequent or less coherent and others are merged if they are determined to overlap significantly. This process was carried out separately by two study team members, in addition to a third reader who was independent of the study team prior to that point, and highly concordant themes across researchers were identified.

Once the themes were finalized, the study team members created a coding scheme to identify the presence or absence of each theme in the mothers’ responses. Study team members experienced in coding qualitative data independently coded sets of 30 interviews to establish reliability. Inter-rater reliability for each theme was assessed using Cohen’s kappa (kappas ranged from .90 - 1.00, indicating excellent agreement). Once inter-rater reliability was established, all of the remaining interviews were coded for the presence or absence of each theme.

### Measures

Additional characteristics of the family, mother and child were collected via questionnaire.

### Measures of family characteristics

Family structure (single parent vs. not) was assessed based on mother’s response to a questionnaire. Family chaos was measured using the Confusion, Hubbub, and Order Scale (CHAOS [24]), a validated and reliable 15-question instrument that measures noise, crowding, disorganization, and confusion in the home (e.g., “You can’t hear yourself think in our home”, ”It’s a real zoo in our home”). Mothers answered “true” or “false” to each item and a summary score was created. Total scores represent the levels of chaos in the home, with higher scores representing more disorganization, confusion and noise (Cronbach’s α = 0.78).

### Measures of maternal characteristics

Mothers reported their own race/ethnicity via questionnaire. Mothers also reported their income from a job over the previous 12 months via questionnaire. We defined maternal employment as a reported maternal income > $0 from a job in the last 12 months. Mothers’ weights and heights were measured without shoes or heavy clothing according to a standardized procedure. Body mass index (BMI) was calculated as weight in kilograms divided by height in meters squared. Mothers’ weight status was categorized as overweight (BMI ≥ 25) or not overweight (BMI < 25). For mothers who were pregnant or had recently given birth, self-reported pre-pregnancy weight was used instead. BMI could not be calculated for two mothers who had given birth within three months of the study visit and did not know their pre-pregnancy weight. Parenting Laxness was measured using The Parenting Scale [25], a reliable and valid self-report questionnaire measure of dysfunctional parenting discipline practices. Mothers indicated their likelihood of responding to child misbehavior with specific discipline strategies in different situations on a seven-point scale (lower scores indicate a tendency to use a more effective strategy and higher scores indicate a tendency to use a less effective strategy). In this study we considered the 11-item Parenting Laxness subscale, given that more permissive parenting has been associated with higher levels of child physical activity [26]. The Parenting Laxness subscale score was calculated as the average of the contributing items (Cronbach’s α = 0.81) and a higher score reflects more permissive parenting.

### Measures of child characteristics

Child date of birth and sex were collected via questionnaire completed by the mother. Child weight and height were measured without shoes or heavy clothing according to a standardized procedure. BMI was calculated as weight in kilograms divided by height in meters squared. Weight status was categorized as overweight (BMI ≥ 85^th^ percentile for age and sex) or not overweight (BMI < 85^th^ percentile) based on the United States Centers for Disease Control and Prevention growth charts [27]. Child temperament, defined as how a child approaches or reacts to the world, was measured using the Child Behavior Questionnaire (CBQ [28]), a widely used and validated [29] maternal report measure of child temperament. In this study we examined four subscales: CBQ Activity Level, CBQ Attentional Focusing, CBQ Inhibitory, and CBQ Impulsivity. CBQ Activity Level (7 items; Cronbach’s α = 0.70) measures the mother’s perception of her “child’s gross motor activity. including rate and extent of locomotion” (e.g., “Seems always in a big hurry to get from one place to another”) [29]. CBQ Attentional Focusing (6 items; Cronbach’s α = 0.71) measures a mother’s perception of her “child’s capacity to maintain attention focus on task-related channels” (e.g., “When picking up toys or other jobs, usually keeps at the task until its done.”) [29]. CBQ Inhibitory Control (6 items; Cronbach’s α = 0.70) measures a mother’s perception of her “child’s capacity to plan and suppress inappropriate approach response under instructions or in novel or uncertain situations” (e.g., “Can lower his/her voice when asked to do so.” [29]. And finally, CBQ Impulsivity (6 items; Cronbach’s α = 0.65) measures a mother’s perception of the “speed of [her] child’s response initiation (e.g., “Usually rushes into an activity without thinking about it.”) [29]. Mothers responded to each item on a seven-point scale (ranging from 1 – “extremely untrue of your child”, to 7 – “extremely true of your child”), with regard to descriptions of the child’s behavior and reactions to certain scenarios and the contributing items were averaged to generate each subscale score. A higher score indicated that the child exhibited more of the temperamental trait.

### Statistical analysis

For the analysis of associations between identified themes and characteristics of the family, mother and child, we built a model predicting each theme being present (vs. not present) using logistic regression. First, we entered both family factors: family structure (single parent vs not) and family CHAOS score, simultaneously into the model. For each of the models, we retained only those family factors that were statistically significant (*p* ≤ 0.05). Next we added all four maternal factors into the model simultaneously: maternal race/ethnicity (White non-Hispanic vs other), maternal weight status (overweight vs not), Parenting Laxness and maternal employment status (employed vs not). Again, we retained only those that were statistically significant. Third, we added the seven child factors simultaneously into the models: child age, child sex, child weight status (overweight vs. not) and child temperament characteristics of CBQ Activity Level, CBQ Attentional Focusing, CBQ Inhibitory Control and CBQ Impulsivity. Again, we retained only those that were statistically significant. Any variable that did not remain significant in this final model was then removed to create the most parsimonious final model.

## Results

### Results of theme about maternal perceptions of PA in their children from semi-structured interviews

Two themes were identified. Illustrative quotes from each theme are shown in Table 3.

**Table 3:** Themes of maternal perceptions of physical activity in their children and supporting quotes

Theme (*n*, %)	Supporting quotes
Theme 1: Mothers believe that their children are active (244, 87.8 %)	*“I think on a scale from 1 to 10, it’s like a 10. It’s very extreme. She’s very active. Now I’m trying to find a sport for her to get into, something to put her into because she’s very, very -- from the time she wakes up to the time she goes to bed -- very, very active. It’s like she never sits down.”*
	*“He really is active. He’s got a lot of energy but I don’t see him as being any different than any other 4- year- old.”*
	*“Very hyper. Very, very hyper. I mean he makes up for all the food that he eats with all the energy that he’s got.”*
	*“Sometimes, he’s wild, he tears around here and … it’s like a zoo and I told him ‘You’ve got to settle down!’”*
Theme 2: Many mothers believe that their children’s high activity level is problematic (75, 27.0 %)	*“Sometimes I will take them someplace where I know they will be running around a lot in order to kind of wear her out a little bit. Like, usually that happens like on a day when she doesn’t have school for whatever reason. So if we’re, or it’s like this outside [motions to snow out the window] it’s all gray and you can’t really do anything.”*
	*“I try to talk to him. I do talk to him. I need to tell him -- and I do tell him that he needs to calm down, sit down -- ‘let’s play like we’re inside not like we’re outside,’ -- but it doesn’t help. It’ll help for a second -- he’s right back at it.”*
	*“I just feel like he’s very energetic and, maybe he needs you know, me to go see a doctor and see if everything’s okay with that because he’s very, very hyper.”*

### Theme 1: Mothers believe that their children are active

The majority (*n* = 244, 87.8 %) of mothers felt that their children were naturally very active, and were not concerned about their children’s activity level. Many mothers related their children’s PA to health and well-being. Mothers often used hyperbole to describe the children’s activity level. Despite their use of hyperbole, mothers also perceived this very high activity level as “normal” or “typical” for a young child.

### Theme 2: Many mothers believe that their children’s high activity level is problematic

Many mothers (*n* = 75, 27.0 %) who thought their children were already active expressed concerns that their children’s activity level so was high that it was problematic. Many verbalized unease with their children’s activity level, describing it as being inappropriate for certain situations and difficult to manage. Mothers expressed concerns that high activity levels could interfere with focus and good behavior; many mentioned concern for attention deficit hyperactivity disorder (ADHD).

Many mothers reported that because they themselves were tired or overwhelmed, or because their children were making a mess, they put significant effort towards reducing their children’s activity level. Mothers would do so by turning on the television, reading a book to the child, or setting up an activity for the child like coloring or puzzles. Few mothers described trying to increase children’s activity level or PA involvement, and when they did so, it was generally to wear the child out with the ultimate goal of reduced activity level.

### Results of associations of characteristics of family, mother and child and themes

The results of the multiple logistic regression models showing the odds of the mother exhibiting each theme based on characteristics of the family, mother and child are shown in Table 4. The theme that mothers feel their children are active (Theme 1) was predicted by the child being younger, child not being overweight and higher CBQ Activity Level subscale scores. Theme 2, mothers feeling that their child’s activity level is problematic, was predicted by lower Parenting Laxness score, the child being male and a lower CBQ Inhibitory Control subscale score. Themes were not predicted by characteristics of the family.

**Table 4:** Logistic regressions predicting presence of themes (*N* = 278)

Theme	1	2
	Mothers believe that their children are active (present vs. not)	Many mothers believe that their children’s high activity level is problematic (present vs. not)
	Odds ratio (95 % Confidence interval)	
Family characteristics		
Single parent household (vs. not)	-	-
Family CHAOS score	-	-
Mother characteristics		
White non-Hispanic (vs. Black non-Hispanic or Other)	-	-
Overweight (vs. not)	-	-
Parenting Laxness	-	0.73 (0.54–1.00)
Employment status employed (employed vs. not)	-	-
Child characteristics		
Age (months)	0.95 (0.90–1.00)	-
Sex (male vs. female)	-	2.78 (1.45–5.26)
Overweight (vs. not)	0.37 (0.17–0.82)	-
Child temperament		
CBQ Activity Level	2.37 (1.59–3.54)	-
CBQ Attentional Focusing	-	-
CBQ Inhibitory Control	-	0.41 (0.30–0.57)
CBQ Impulsivity	-	-

## Discussion

This study makes new contributions to and supports some previous findings in the literature. First, like other studies [8–12, 16] in Canadian, European and Australian cohorts, we found that US mothers viewed their young children as already very active, and were generally not concerned about their children’s activity level. This theme was predicted by the child not being overweight, and by having a more active temperament. This is similar to the findings of Corder et al. [30], who found that maternal overestimation of child activity levels was associated with lower child fat mass indices. Mothers of children with overweight in our study were less likely to have this theme, possibly because they may be accurately perceiving their overweight children’s need for more PA. Alternatively others [2] have described that mothers believe that overweight causes inactivity and not the reverse, which may be what mothers are expressing. Second, this study found that mothers often described their children’s activity level as problematic. While others [16] have reported mothers trying to reduce their children’s activity level, no previous study, to our knowledge, has reported high PA as problematic. This theme was predicted by lower Parenting Laxness, the child being male and having lower CBQ Inhibitory Control. This finding is consistent with the literature supporting both higher measured [31, 32] and parentally perceived [8] PA levels in males.

The finding that many mothers perceived their children as already very active leads to some challenges regarding how pediatric providers can best deliver physical activity promotion messages to parents of young children. Mothers may be overestimating their children’s PA level, as has been previously reported [30, 31]. In this case, the pediatrician must persuade a mother to encourage greater activity levels in a child she already perceives as active [16]. Alternatively, as others have reported [33], mothers may accurately perceive that their children are extremely active. In this case, efforts may be best focused on identifying practical outlets in the children’s homes and communities for their high activity needs.

Whereas it is developmentally appropriate for young children to be energetic and active, the observation that many mothers found their children’s daily activity level to be problematic has not, to our knowledge, been reported previously in the physical activity and health promotion literature. The finding that mothers also reported putting more effort into decreasing rather than increasing their children’s activity level is important, as these mothers may be less inclined to promote PA in their children. This theme was associated with lower Parenting Laxness, or less permissive parenting. These mothers who are stricter and more consistent in providing discipline are more likely to be bothered by their child’s high PA level. Mothers also described in Theme 2 concern that over-activity interfered with behavioral compliance and ability to focus; this finding adds additional insight into reasons why mothers may believe that a high activity level is problematic. Prior research [12], via focus group analysis, has described how parents may perceive PA as displacing “creative and mental pursuits” in their children. Mothers may be concerned that high activity levels may be associated with decreased school readiness or detract from academic achievement in their children.

The perception of high PA level being problematic is concerning for several reasons. First, as mentioned above, these mothers may be less inclined to want to increase PA in their children, and may, in fact, devote more time to decreasing their children’s activity to a more manageable level. Secondly, this perceived over-activity may be difficult for mothers to manage, leading to harsh parent–child interactions, which may lead to a negative perception of PA among the children and a preference for sedentary activities. Lastly, promotion of PA in children can lead to improved behavioral outcomes at school [34, 35] and improved executive function [36], with less desirable behavior associated with restricting PA [37]. Thus, although promotion of PA in a child who is already perceived as overly active may seem counterintuitive to many mothers, explaining that it may in fact be beneficial for behavior at home and school may be a possible PA promotion strategy.

Strengths of this study include a large sample size, inclusion of characteristics of the family, inclusion of both mother and child characteristics in our analysis, as well as the semi-structured interview format. Several limitations should be considered. The mothers’ answers to the questions about physical activity may have been affected by the series of questions that preceded them. There may also have been social desirability bias in their answers leading to a greater number of mothers who described their child as very active. Furthermore, children’s actual physical activity levels were not measured, therefore it cannot be determined how closely maternal perceptions of child activity level align with objective measures. In addition, only low-income English speaking mothers were included in this sample. Ultimately, the results of this qualitative work may only be applicable to other native English speaking low-income mothers in this area of the US. Finally, although complex and difficult to measure, parenting is likely an important contributor to child PA. Future works should include more detailed and observational measures of parenting styles and practices, to test their associations with maternal beliefs about child activity level.

## Conclusions

Mothers believe their young children to be sufficiently active, and sometimes overly active. Public health messages about promoting PA in young children may be made more effective if they are informed by a more detailed understanding of how mothers of young children think about PA. Future work might consider testing the effectiveness of incorporating these maternal belief systems into a childhood obesity intervention designed to increase maternal promotion of young children’s physical activity.

## Abbreviations

PA: Physical activity
US: United States
CHAOS: Confusion, hubbub and order scale
BMI: Body mass index
CBQ: Child behavior questionnaire
ADHD: Attention deficit and hyperactivity disorder
